# Floating gallbladder identified on preoperative computed tomography and managed laparoscopically: a case report

**DOI:** 10.1093/jscr/rjag360

**Published:** 2026-05-13

**Authors:** Shumarova Svetlana, Miteva Irina, Sokolov Manol

**Affiliations:** Department of Surgery, University Hospital “Aleksandrovska”, Medical University, 1431 Sofia, Bulgaria; Medical University, 1 Georgi Sofijski Blvd, 1431 Sofia, Bulgaria; Department of Surgery, University Hospital “Aleksandrovska”, Medical University, 1431 Sofia, Bulgaria; Medical University, 1 Georgi Sofijski Blvd, 1431 Sofia, Bulgaria; Department of Surgery, University Hospital “Aleksandrovska”, Medical University, 1431 Sofia, Bulgaria; Medical University, 1 Georgi Sofijski Blvd, 1431 Sofia, Bulgaria

**Keywords:** floating gallbladder, laparoscopic cholecystectomy, anatomical variation, incidental finding

## Abstract

Floating gallbladder is a rare anatomical variant characterized by absence or marked attenuation of its attachment to the hepatic bed, allowing increased mobility within the peritoneal cavity. Although this condition is considered a predisposing factor for gallbladder torsion, it may remain clinically silent and is often identified incidentally during surgery or preoperatively with computed tomography. Preoperative diagnosis remains challenging despite advances in imaging techniques. We report the case of a 73-year-old male patient admitted for elective laparoscopic cholecystectomy due to symptomatic cholelithiasis. Preoperative imaging showed chronic calculous cholecystitis and lack of communication between the gallbladder and liver. A safe laparoscopic cholecystectomy was performed. Gallbladder mobility may influence intraoperative orientation and increase the risk of biliary misidentification. Laparoscopic cholecystectomy remains a safe and effective treatment when meticulous dissection and identification of anatomical structures are achieved.

## Introduction

A floating gallbladder is an uncommon anatomical variant in which the gallbladder lacks its normal attachment to the hepatic bed and is instead suspended by an elongated cystic pedicle, allowing abnormal mobility within the peritoneal cavity. This increased mobility has been described as a predisposing factor for torsion and vascular compromise, although in many cases, the condition remains clinically silent and is discovered incidentally during imaging or surgery [1].

The exact prevalence of a completely free-floating gallbladder is difficult to determine because most available evidence consists of isolated case reports and small case series rather than large cohort studies. Nevertheless, anatomical and radiological reviews suggest that while minor variations in gallbladder mobility may occur in a small proportion of the population, a true floating gallbladder is extremely rare and is most often identified intraoperatively [2].

Most reported cases occur in elderly patients, and several authors have proposed that age-related reduction in visceral fat, elongation of mesenteric attachments, and hepatic atrophy may contribute to increased gallbladder mobility. These changes may explain why the condition is more frequently described in older individuals and has been reported with a slight female predominance [3].

In this context, we report a case of an incidentally discovered true floating gallbladder identified during elective laparoscopic cholecystectomy in an elderly patient.

## Case presentation

A 73-year-old male patient was admitted to the Department of Surgery at Alexandrovska University Hospital, Sofia, for elective operative management of symptomatic cholelithiasis. The patient reported recurrent biliary colic characterized by episodic right upper quadrant pain radiating to the right scapular region over several months, with increasing frequency prior to admission.

His medical history was significant for arterial hypertension, bronchial asthma, osteoporosis, and antineutrophil cystoplasmic antibodies–associated vasculitis (granulomatosis with polyangiitis) treated with long-term immunosuppressive therapy, including corticosteroids and rituximab. He also had chronic kidney disease in the setting of congenital right renal agenesis.

On admission, physical examination of the patient revealed mild right upper quadrant tenderness without guarding or peritoneal signs. Laboratory investigations demonstrated leukocytosis (20.2 × 10^9^/l) with neutrophilia, mild anemia, elevated gamma-glutamyl transferase levels, and impaired renal function consistent with known chronic kidney disease. Liver transaminases and bilirubin levels were only mildly elevated. Leukocytosis is most likely associated with vasculitis as a concomitant disease and superimposed cholecystitis.

**Figure 1:**
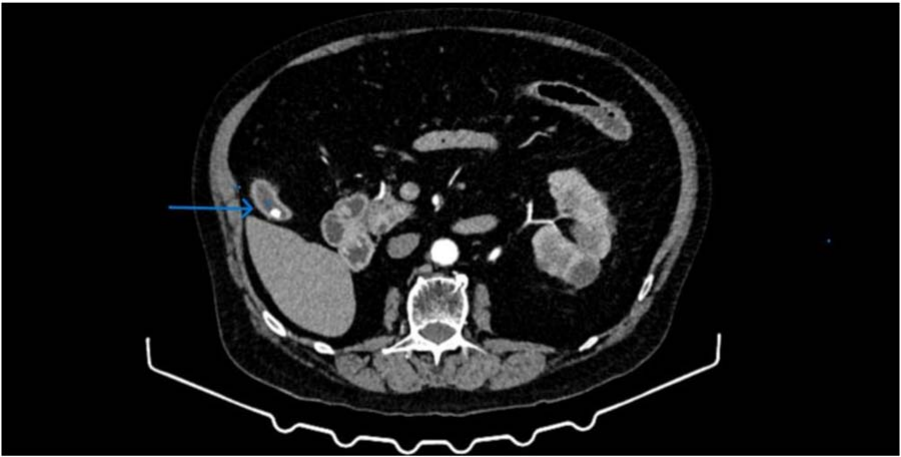
CT showed the presence of a gallbladder calculus as well as a lack of communication between the gallbladder and the hepatic parenchyma.

Contrast-enhanced computed tomography (CT) revealed a contracted gallbladder with thickened walls and a calcified stone, consistent with chronic calculous cholecystitis (Fig. 1). Notably, the gallbladder appeared separated from the hepatic parenchyma, without its usual hepatic attachment, suggesting a floating gallbladder configuration (Fig. 1). The intra- and extrahepatic bile ducts were not dilated, and no radiological signs of acute inflammation or perforation were identified.

Following multidisciplinary assessment, including cardiology, nephrology, and anesthesiology consultation, the patient was considered suitable for elective laparoscopic cholecystectomy with moderate operative risk.

**Figure 2:**
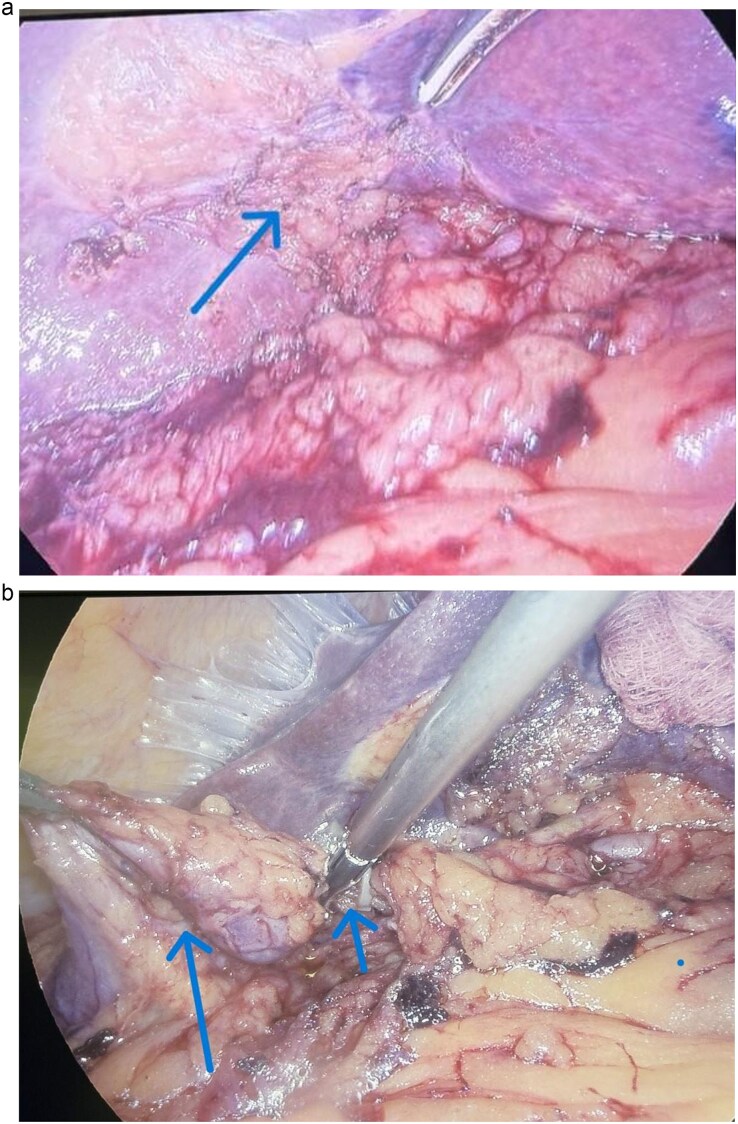
(a) Intraoperative findings: Absence of attachment to the hepatic parenchyma and of the presumed gallbladder fossa was observed. (b) Intraoperative findings: A floating gallbladder was observed, along with the clips applied to the cystic artery and cystic duct.

Laparoscopic cholecystectomy was performed under general anesthesia using a standard 4-port technique. Exploration of the right upper quadrant revealed an unusual anatomical finding: the gallbladder was markedly atonic with thinned walls and was completely free-floating within the peritoneal cavity, lacking normal attachment to the hepatic bed. No peritoneal fixation to the liver parenchyma was present (Fig. 2a). The organ was connected only by an elongated cystic pedicle containing the cystic duct and cystic artery (Fig. 2b). Omental adhesions were noted and carefully dissected. There were no signs of torsion, ischemia, gangrene, or perforation. The gallbladder was freely mobile within the subhepatic space, consistent with a true floating gallbladder. Meticulous dissection was undertaken to achieve the critical view of safety. The cystic duct and artery were clearly identified, clipped, and divided. Laparoscopic cholecystectomy was completed without intraoperative complications, and a subhepatic drain was placed.

The postoperative course was uneventful. The patient recovered well, resumed oral intake early, and remained clinically stable. He was discharged on postoperative day 3 in good condition. Histopathological examination revealed chronic hyperplastic calculous cholecystitis with cholesterolosis. Microscopy demonstrated mucosal hyperplasia, Rokitansky–Aschoff sinuses, and chronic inflammatory infiltrates within the lamina propria.

## Discussion

A floating gallbladder is a rare anatomical variant in which the gallbladder lacks normal fixation to the hepatic bed and is instead supported only by an elongated cystic pedicle, allowing increased mobility within the peritoneal cavity. This anatomical configuration is widely considered the main predisposing factor for gallbladder torsion, although torsion develops only in a minority of patients [4]. Gallbladder torsion itself remains extremely uncommon, with an estimated incidence of approximately 1 in 300 000–400 000 hospital admissions. Because torsion represents the most severe clinical manifestation of a floating gallbladder, most published cases describe acute presentations with ischemia, necrosis, or perforation. In contrast, incidental intraoperative identification of a freely mobile gallbladder without torsion appears to be reported less frequently [3]. The present case falls into this latter category, as the gallbladder was found to be completely free-floating during elective laparoscopic cholecystectomy but showed no evidence of rotation, ischemia, or vascular compromise.

Several mechanisms have been proposed to explain the development of a floating gallbladder. Congenital persistence of a mesentery may prevent normal fusion of the gallbladder to the liver during embryologic development. In addition, acquired factors such as loss of visceral fat, hepatic atrophy, and elongation of supporting structures in elderly patients may further increase organ mobility [2]. These mechanisms may explain why most reported cases occur in older individuals. Our patient, although male, belonged to the typical age group in which such anatomical changes have been described in the literature. While some series suggest a female predominance, cases in male patients have also been reported, indicating that age-related anatomical changes may be more relevant than sex alone [1].

Preoperative diagnosis of a floating gallbladder remains difficult. Imaging findings are often nonspecific, and absence of hepatic attachment may not be clearly demonstrated even on CT or ultrasonography. As a result, the diagnosis is frequently established intraoperatively [5]. Similar diagnostic challenges have been described in recent case series in which floating gallbladders were recognized only during surgical exploration [1]. In the present case, preoperative imaging demonstrated chronic calculous cholecystitis as well as lack of usual attachment to the liver parenchyma.

From a surgical perspective, awareness of this anatomical variation is important. A freely mobile gallbladder may alter the orientation of structures within the hepatocystic triangle and theoretically increase the risk of biliary misidentification if the anatomy is not carefully assessed. Therefore, it is essential to clarify the anatomy preoperatively, if possible, using imaging methods such as CT, magnetic resonance imaging. In our patient, CT showed a lack of normal liver fixation and laparoscopic cholecystectomy was completed safely using a standard four-port technique after careful identification of the critical view of safety. This experience is consistent with recent reports indicating that laparoscopic management is feasible and safe when meticulous dissection is performed and anatomical structures are clearly identified [6].

## Conclusion

Floating gallbladder is a rare anatomical variant that may show no clinical symptoms and is often identified incidentally during surgery. Although most reports emphasize its association with torsion, this condition can also be encountered as an incidental finding during elective cholecystectomy. Awareness of this variation is important for surgeons, since altered gallbladder mobility may affect intraoperative orientation and increase the risk of biliary misidentification. Careful dissection and attainment of the critical view of safety allow laparoscopic cholecystectomy to be performed safely even in the absence of normal hepatic fixation.

## Data Availability

The datasets used during the current study are available from the corresponding author upon request.
